# Effect of electronic health records on doctor-patient relationship in Arabian gulf countries: a systematic review

**DOI:** 10.3389/fdgth.2023.1252227

**Published:** 2023-10-06

**Authors:** Celine Tabche, Mays Raheem, Arwa Alolaqi, Salman Rawaf

**Affiliations:** WHO Collaborating Centre, Department of Primary Care and Public Health, Imperial College London, London, United Kingdom

**Keywords:** electronic health records, electronic medical records, information systems, computer records, EHR systems, GCC

## Abstract

**Background:**

The electronic health record (EHR) has been widely implemented internationally as a tool to improve health and healthcare delivery. However, EHR implementation has been comparatively slow amongst hospitals in the Arabian Gulf countries. This gradual uptake may be linked to prevailing opinions amongst medical practitioners. Until now, no systematic review has been conducted to identify the impact of EHRs on doctor-patient relationships and attitudes in the Arabian Gulf countries.

**Objective:**

To understand the impact of EHR use on patient-doctor relationships and communication in the Arabian Gulf countries.

**Design:**

A systematic review of English language publications was performed using PRISMA chart guidelines between 1990 and 2023.

**Methods:**

Electronic database search (Ovid MEDLINE, Global Health, HMIC, EMRIM, and PsycINFO) and reference searching restricted to the six Arabian Gulf countries only. MeSH terms and keywords related to electronic health records, doctor-patient communication, and relationship were used. Newcastle-Ottawa Scale (NOS) quality assessment was performed.

**Results:**

18 studies fulfilled the criteria to be included in the systematic review. They were published between 1992 and 2023. Overall, a positive impact of EHR uptake was reported within the Gulf countries studied. This included improvement in the quality and performance of physicians, as well as improved accuracy in monitoring patient health. On the other hand, a notable negative impact was a general perception of physician attention shifted away from the patients themselves and towards data entry tasks (e.g., details of the patients and their education at the time of the consultation).

**Conclusion:**

The implementation of EHR systems is beneficial for effective care delivery by doctors in Gulf countries despite some patients' perception of decreased attention. The use of EHR assists doctors with recording patient details, including medication and treatment procedures, as well as their outcomes. Based on this study, the authors conclude that widespread EHR implementation is highly recommended, yet specific training should be provided, and the subsequent effect on adoption rates by all users must be evaluated (particularly physicians). The COVID-19 Pandemic showed the great value of EHR in accessing information and consulting patients remotely.

## Background

Continuity of patient care and overall healthcare safety are strongly associated with reliable medical records. Traditionally, medical records have used paper-based systems to record relevant details such as treatment and outcomes. However, in recent years, medical organisations are increasingly turning to computerisation for managing the plethora of data surrounding each patient entering the health system. The electronic health record (EHR) is a promising tool for enhancing national and international healthcare, particularly primary care delivery (1). An EHR can be defined as an application environment that captures the individual clinical data of patients, is linked to a clinical decision support system, and allows computerised order entry and clinical documentation applications (2). Electronic health records were initially developed and used at academic medical facilities, but many leading providers in the health industry are implementing computerised clinical record systems to manage the huge volume of clinical, administrative, and regulatory information that occurs in contemporary health care (3). The COVID-19 Pandemic and the need for virtual care demonstrated how essential EHRs are in delivering effective care remotely in both primary and hospital care settings.

Introducing any new information technology system into an organisation leads to changes in processes and workflows, which can lead to user dissatisfaction as they encounter teething problems in the system, bugs, or even simple annoyance at the requirement to learn a new way of doing things (4). Although EHRs are gradually entering widespread use within the Arabian Gulf, their impact on physician attitudes and the patient-doctor relationship remains to be determined. A previous systematic review has examined the impact of EHRs on patient-physician communication, finding no significant change in patient satisfaction or patient-doctor communication (5). However, this work focused on Western (Europe, United States, Australia) medical facilities; thus, the relevance to the culturally differing healthcare systems of the Gulf countries is still to be seen.

To shed light on this field, this systematic literature review seeks to understand the impact of EHR use on patient-doctor relationships and communication in the Gulf Cooperation Council (GCC) countries.

## Methods

### Search strategy

A systematic review follows the Preferred Reporting Items for Systematic Reviews and Meta-Analyses (PRISMA) standards.

It was performed based on the methods of Alkureishi et al. (5). This study conducted a systematic electronic search of the English literature in Ovid MEDLINE, HMIC (Health Management Information Consortium), Eastern Mediterranean Index Medicus (IMEMR), Global Health, and APA PsycINFO between December 2021 and Sep 2023, with no date limit. Moreover, cited references searching of prior reviews and a manual search were performed with Scopus, Google Scholar, PubMed and The Cochrane Library.

The search on all databases was ((“electronic w/1 record”) OR (“EMR system”) OR (“computer w/1 record”) OR (“Medical Records Systems, Computerized”)) AND ((“middle AND east”) OR (“gulf AND cooperation AND council”) OR (“GCC”) OR (“KSA”) OR (“UAE”) OR (“saudi AND arabia”) OR (“Oman”) OR (“Bahrain”) OR (“Qatar”) OR (“Kuwait”) OR (“united AND arab AND emirates”) OR (“arab AND states”)).

The systematic search included all relevant studies by exploring Medical Subject Heading (MeSH) terms and keywords related to electronic health records and doctor-patient communication and relationship. The selection of search terms was performed in consultation with a biomedical librarian.

To explore publication bias and mirror Alkureishi et al. (5), unpublished studies were searched for in past meeting abstracts of the Society of General Internal Medicine, the American Academy of Family Physicians, the International Conference on Communication in Healthcare and the European Association of Communication in Healthcare.

Only studies related to EHR use, and face-to-face patient-doctor communication or relationship were included; editorials and commentaries were excluded. All study designs, and all patient populations were included, but the search was restricted to studies on the six Gulf countries (Bahrain, Kuwait, Oman, Saudi Arabia, United Arab Emirates). Studies exclusively reported physician attitudes, perceptions, and other interactions, but face-to-face patient-doctor relations (i.e., remote online access) were discarded.

### Study selection

After duplicate removal, title and abstract screening were performed by the author and an independent reviewer. When titles or abstracts were unclear, they were included in full-text screening, followed by a second independent review. Quality assessment of the final selection was performed using the Newcastle-Ottawa Scale (NOS).

### Data synthesis and analysis

Studies were compared by the study population, design and outcomes and sorted according to the data collection method. They were then organised in a data extraction table. Initial codes relating to the research question were generated to capture the ideas from the studies. Themes were defined from recurring patterns that provided insights into the effects of EHR implementation on patient-doctor interactions.

The data collected did not allow for a meta-analysis due to the heterogeneity in interventions, methodology, and outcomes of the studies included. Thus, a narrative synthesis was conducted.

**Figure 1:**
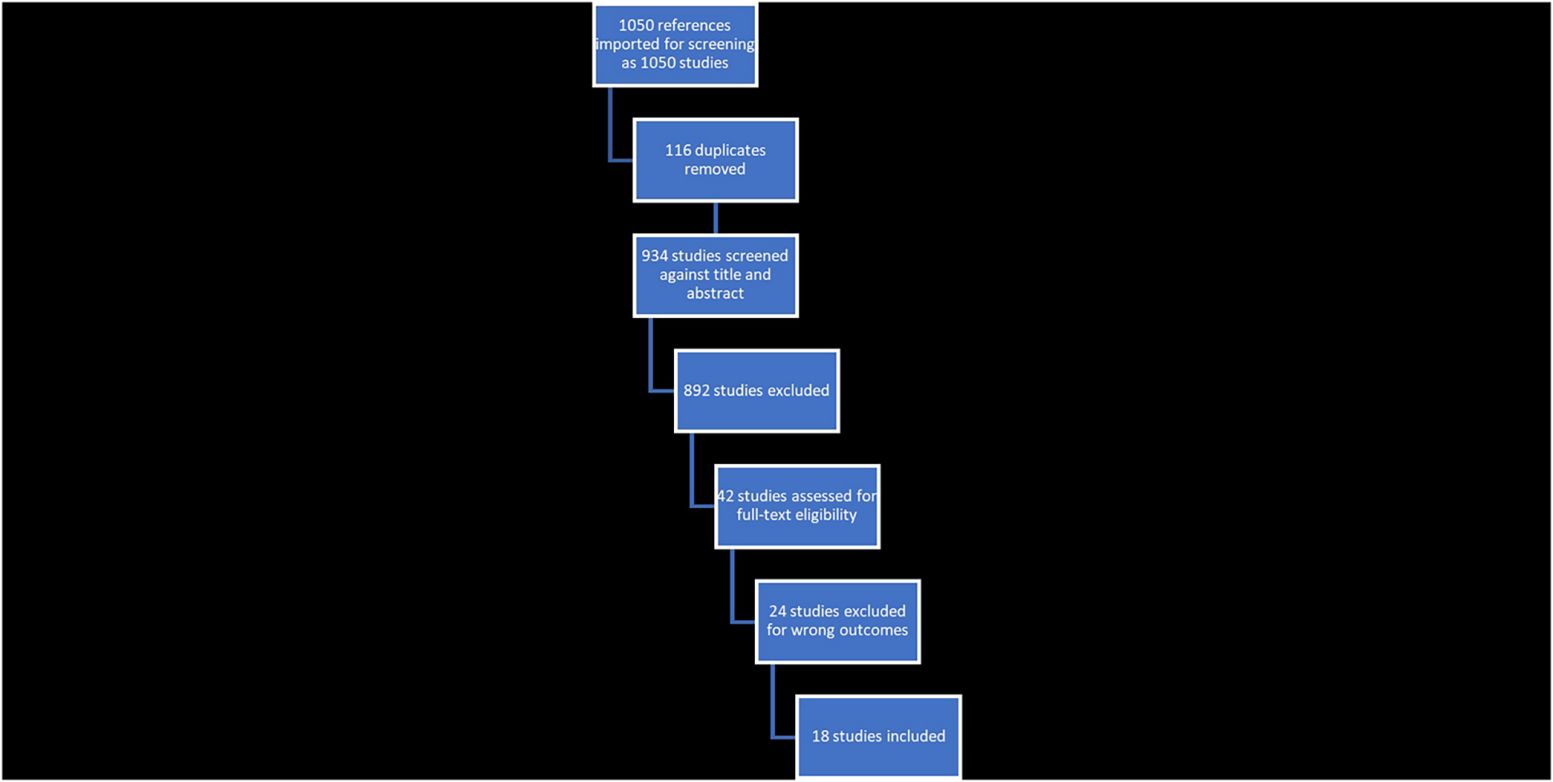
Exported PRISMA data from covidence.

The initial search provided a total of 94 studies. From these, 93 studies remained for screening following the removal of duplicates. Manual search and backward reference screening produced a further 7 items. Seventy studies were excluded in an initial assessment (e.g., references lacking an abstract or unrelated to health). A detailed analysis of the remaining 30 studies was performed. As part of this detailed analysis, 15 studies were excluded as irrelevant to electronic health records or not presenting a doctor-patient relationship outcome. The 18 remaining studies were then included. An overview of this process is shown in Figure 1.

### Data availability

The data supporting this systematic review are from previously reported studies and datasets. Details of the publications used in this systematic review are included in Table 1.

**Table 1:** Summary of the studies extracted that show the relationship between doctors and patients when using the EMRs.

Date/Author	Title	Methodology	Primary outcomes	Secondary outcome
1-Al Farsi (6)	Use of Electronic Medical Records in Oman and Physician Satisfaction	Nonexperimental “survey” research, Included 70 physicians Age of physician is from 30 to 65 years. Included both female and Male physicians.	EMR improves the communication between department in 95% Improves the quality care of patient (85%); Retrieval and entry of patient information is accurate and easy way (80%) Access to the system is easy and available (70%) Reduces medical errors (67%) Enhances the productivity (59%).	80% agreed the EMR system required increased confidentiality. The EMR system is underutilized (75% of physicians agree); hospital is still using a paper system in some departments (74%); Disease coding represents a major problem (70%); The EMR system is time consuming (67% agree); Speed of the system is too slow (60%). Participants’ feedback: Each doctor receives a specific access password that changes periodically. // Train and advise staff on the requirements of patient // Information privacy and encourage their input on how protection should be developed.
2-Al-Azmi et al. (7)	Patients’ Satisfaction with Primary Heath Care in Kuwait After Electronic Medical Record Implementation	Descriptive cross-sectional A random sample of 200 people (at age of 18 years and above) Structured questionnaire Exit interview (47.5%) were males and 52.5% were females	81.5% of them agreed that EMRS improved the physician performance. System helped to improve not only arrangement of patient's turn (93.0%), but dispense medication from pharmacy (92.0%) Improved accuracy and easiness of follow up for health status was 89.5% System improved time spent to retrieve medical record (78.5%) and that for receiving medication (82.0%).	Physician selection or waiting time. The system did not achieve much improvement in physician selection (29.5%) or waiting time (25.5%). 13.2% of the participants did not like physician's handling of computers in the examination room as this decreases his attention and communication.
3-Al-Mujaini et al. (8)	Satisfaction And Perceived Quality of An EMR System in A Tertiary Hospital in Oman	A cross-sectional survey including 200 physicians in Oman over 6 months. Response rate was 70.5% (141/200) Of the 141 respondents; 55 (39%) were consultants, 28 (19.9%) were registrars and 21 (14.9%) were senior house officers (SHO). Eighty-one (57.4%) participants were males. The majority of the participants were in the third to fourth decade of age.	(15.4%) Respondents rated the EMR to be a success in improving their work in terms of quality, performance, and timelines Factors affecting poor ratings: (OR = 1.3 95% 0.2 7.3) not working with patients (15.6%) rated the current EMR system as an effective tool.	29.4% of respondents considered EMR not worth the time and effort required to use it. In our study, the low satisfaction associated with junior designations and respondents with low familiarity with computers may be related to this fact (67.4%) Reported increasing difficulty with the performance of work after applying the EMR system. The overall quality of work was perceived not to have changed (41.2% of the respondents) or declined (27.4% of the respondents). 60% of users still resorted to paper records for some tasks not supported by EMR, such tasks included statistics, codification, and patient transfers between institutions. difficulty in transferring older paper-based records to the EMR system, issues based on long-term preservation and storage of data, as well as how to ensure the physical and virtual security of the archives, software problems of codification (standards that help to ensure that clinical information input and retrieval are not arbitrary), and customization (system adapted for the users and tailored to workflows specific to a user site) (68.1%) had prior training on the use of the EMR system
4-Al-Jafar (9)	Exploring Patient Satisfaction Before and After Electronic Health Record (EHR) Implementation: The Kuwait Experience	A random sampling 700 subjects The majority of participants (67 percent) were 19 to 34 years of age.	EHRs increase trust in doctors 27% Doctor pays more attention to typing, and EHRs increase trust in physicians) varied from 36 to 50 percent EHRs affect relationship with doctors 27%	At visit, doctor pays more attention to typing 17%
5-Al-Rowibah et al. (10)	The Impact of Computerized Physician Order Entry on Medication Errors and Adverse Drug Events.	Cross-sectional research design at the King Fahad Medical City Hospital (KFMCH) of the Kingdom of Saudi Arabia (KSA). 93 physicians	88% of the physicians agreed that the use of CPOE improved their performance 76% reported that the use of CPOE increased their productivity 56% of the participants agreed that CPOE was a simple system 64% reported that it was easy to use.	44 percent of the physicians agreed that CPOE lacked a user guide during medication ordering
6-Al Alawi et al. (11)	Physician User Satisfaction with an Electronic Medical Records System in Primary Healthcare Centres in Al Ain: A Qualitative Study	A total of 23 physicians—focus groups A descriptive qualitative study conducted in primary healthcare centres (PHC) in Al Ain, United Arab Emirates (UAE).	Physicians expressed satisfaction with the orders and results of laboratory and radiology functions Physicians satisfied with the electronic prescription function, stating that it reduced errors and saved time. Physicians’ perceptions about patient reaction were mixed. unhappy because of the disturbed patient–doctors’ relationship waiting time increased due to data entry causing more frustration to the patients. Difficulty in use at the beginning ▸ Training was sufficient and good Past computer skills ▸ Different users’ generations with different, computer skills The impression about the pre-completed notes ▸ Precompleted notes definitely saves time Doctor–patient relationship • No eye contact • Waiting time is more • Patients are accepting the system because it is reflecting an advance modern of technology. Many physicians were concerned about their patients’ perception about the new technology. They felt that many patients were unhappy but indicated that few patients approved and made positive remarks to their physicians.	The impression about the pre-completed notes and no eye contact Many physicians were pleased about the orders and results of laboratory and radiology as they emphasised that this is the strongest point in the EMR system. Participants identified the messaging system within the EMR software as a practical, useful, and important tool for enhancing efficiency within the team. Major barriers to implementation and adoption included computer literacy, training, and time. Participants mentioned the loss of confidentiality in the patient's files, because anybody who has access could open any file.
7-Alasmary et al. (12)	The Training on Clinical Productivity and User Satisfaction In Using The Electronic Medical Record In Saudi Arabia	123 physicians and nurses A cross-sectional study design	Self-reported system productivity and satisfaction was statistically correlated at *p* < 0.01 (*R* = 0.509)	?
8-Alharthi et al. (13)	Physician Satisfaction with Electronic Medical Records in a Major Saudi Government Hospital	220 physicians—115 included 71% were male, 64% were Saudi, Cross-sectional analytical observational study Average age was 39.8 years. Government hospital in the Eastern Province, Kingdom of Saudi Arabia The tool used to collect the data was a self-administered survey based on the DeLone and McLean model	Only 40% were satisfied with the system; furthermore, 61% were willing to totally abandon the system and go back to paper records. Of the physicians surveyed, 90%wanted to change the system, and 70% of those who did not want to go back to a paper system wanted to change this system. 60%of the physicians agreed with Information is accurate”, “relevant” About 50% agreed with the statements “features allow me to perform my work well” and “the performance of the system is reliable” “system easy to use” and “security is acceptable”, were satisfactory to about 65% Fewer than half of the respondents agreed “the system is fast” and “the system is integrated with my workflow”.	Only 48% of the system was integrated well with their workflow The physicians in our study might also have been experienced computer users but expected more sophisticated training. Additionally, only half found that the system support was acceptable. The screen layout was acceptable to 62% of the physicians, who considered that information was presented in a suitable format. The physicians were not satisfied with the completeness or accuracy of the information: only 45% reported that the information was complete and 64% that it was accurate. The system was considered easy to use by 64% of the physicians, a finding similar to those of other studies. 58% of the physicians were dissatisfied with the speed of the system, reporting that it took a long time to move between screens and that the system was slow to start up.
9-Shaker et al. (14)	Physicians’ Perception About EMR System in Makkah Region, Saudi Arabia	Cross-sectional survey 317 completed questionnaires, Age group 36–45 years Subjects were from King Fahd Hospital	Improved quality of practice was appreciated by majority, that is, 77.7% “EMRS improve quality of practice (work life)” 71.2%,	“It does not disrupt the workflow” (35.1%) “EMRS is comfortable while entering the data instead of writing” (34.8%)
10-Bani-Issa et al. (15)	Satisfaction Of Health-Care Providers with EMR And Perceived Barriers To Its Implementation In The United Arab Emirates	A cross-sectional survey of 680 health-care providers	Of the 680 participants, a total of 317 (46.6%) expressed high levels of satisfaction with the EHR system	A lack of trust in the system's reliability Inadequate computer skills and inadequate training
11-Alsohime et al. (16)	Satisfaction And Perceived Usefulness with Newly Implemented Electronic Health Records System Among Paediatricians At A University Hospital	A cross-sectional survey [112 physicians who completed the survey, 97 (86.6%) attended training courses before the implementation of new HER] distributed to all physicians working in the paediatric department of King Saud University Medical City (KSUMC)	The participants rated the perceived usefulness of the new system at 6.4/10 for patient care and physicians’ satisfaction levels were 5.2/10. The top indicator of HER usefulness was the system's ability to reduce errors and improve the quality of care [mean 3.31, SD 0.9, RII 82.8%]; The top indicator of satisfaction with the HER system was enhanced “individual performance” [mean 3.04, SD 1, RII 60.9%];	This study revealed that higher level of satisfaction was associated with the perceived positive effect of HER on individual performance and patient care. The results of this study are provided a “pediatricformatics” insight to establish specialized children hospital and similar forms of integrated paediatric health care services. Academic centres might face other difficulties in implementing HER, due to the involvement of medical students, interns, and residents in the clinic care
12-Al-Hashimi et al. (17)	The Potential For Medical Informatics In The Eastern Mediterranean Region	A feasibility study questionnaire done in December 1989 at Salmaniya Medical Centre (SMC) in the state of Bahrain.	Opinions about the current information system. Twelve of the physicians (22.2%) indicated that 30% of their time is spent on daily charting and paperwork. Knowledge about computer usage. Only 17 physicians (32.1%) had worked with computers before. Most of the physicians either “strongly agree” or “agree” (46/86.8%) that computerization will make some of the physician's duties easier Ranking of the priority of certain tasks to be computerized. The majority of the physicians ranked generating clinical reports not less than an 80% priority (38/74.5%).	Based on this study, the HIS experience in Bahrain will be different from the experience in the United States due to some factors: (a) the health care system in Bahrain is centrally organized under the Ministry of Health, b) the majority of physicians in Bahrain support the idea of HIS, based on the idea that the system will help them to optimize patient care and save time c) the majority of physicians feel that they are under pressure from increased patient flow and limited bed capacity, d) the majority of physicians believe that there is a need for more specialized physicians. One final contrast between this evidence from Bahrain and the United States is that the acceptance and diffusion of HIS among physicians in the United States was slow from the start.
13-Khalifa (18)	Perceived Benefits of Implementing And Using Hospital Information Systems And Electronic Medical Records	The study used quantitative survey methods through a questionnaire to collect data and information directly from different categories of healthcare professionals of four Saudi hospitals. Data was collected with 153 valid responses out of 300 selected participants	Participants overall agree that there is a lot of benefit from using Hospital Information system and Electronic medical records (the mean was 4.18) Participants strongly agree that the HIS and EMR improve the information access (mean was 4.49) Participants strongly agree that the HIS and EMR Increased healthcare professional Productivity (mean was 4.31) Participants strongly agree that the HIS and EMR improved efficiency and accuracy of coding and billing (mean was 4.30) Participants strongly agree that the HIS and EMR improved quality of healthcare (mean was 4.27) Participants strongly agree that the HIS and EMR Improved clinical management (mean was 4.22)	HIS and EMR can also decrease costs of some services such as medical transcription and reporting, making the work of healthcare professionals more productive.
14-Saddik and Al-Fridan (19)	Physicians’ Satisfaction with Computerised Physician Order Entry (CPOE) At the National Guard Health Affairs: A Preliminary Study	A survey was developed measuring physician satisfactions with CPOE on Likert scale.	85% of the physician perceived that the CPOE reduced patient care errors and that the order system easy to use. 50% of physician reported a positive and negative perception that Laboratory data retrieval is fast. 60% of the physician stated that they were satisfied with order entry system.	The study shoes that COPE characteristics were strongly correlated to physician satisfaction efficiency and quality of care.
15-Wali (20)	Patient Satisfaction with The Implementation of Electronic Medical Records In The Western Region, Saudi Arabia, 2018	A cross-sectional survey was conducted to explore patient satisfaction with the EMR compared to the previous PMR in 2018 in PHCs in Jeddah, KSA. The setting was the waiting areas in the PHCs, all are satellite clinics of King Abdulaziz Medical City in the Western Region, including Jeddah, Makkah, and Taif. The study was conducted over 6 months, from July 10 to December 31, 2018. The centers are Bahra, the Specialized Polyclinic, King Faisal Residential City Clinic in Jeddah, Sharia Primary Healthcare Clinic in Makkah, and King Khalid Residential City Clinic in Taif. 377 participants	physician's attention to the patient during the consultation improved from 77% (*n* = 291) to 82.3% (*n* = 314) with the implementation of EMR and the physician's explanation of the reasons for ordering tests and medication improved from 80.7% (*n* = 302) to 85.8% (*n* = 325). The time spent with the patient during the consultation also improved from 73.8% (*n* = 279) to 80.4% (*n* = 303) and active listening improved from 73.5% (*n* = 278) to 77.3% (*n* = 289). Patients feeling that the physician is more interested in the medical records improved from 44.1% (*n* = 166) to 57.5% (*n* = 218) majority agreed that the implementation of the EMR improved the physician-patient relationship in general and reduced the waiting time (63.9%, *n* = 242). The majority (81.6%) also agreed that the services provided by the PHC im- proved with the implementation of EMR; specifically, more efficient prescription dispensing (80%, *n* = 304), improved appointment booking (80.6%, *n* = 304) and an improved referral system (76.1%, *n* = 287)	This study showed positive participant's perception about primary health care services after implementation of the EMR in all of the aspects; it improved information access, health care professional productivity, quality of provided health care, and overall patient satisfaction, overall physician-patient relationship improved with the implementation of EMR, as well as the total waiting time and the overall quality of services, the appointment booking time, and referral system.
16-Karim Jabali (21)	Predictors Of Anaesthesiologists’ Attitude Toward Ehrs in Saudi Arabia For Clinical Practice	Cross-sectional study, carried out in Saudi Arabia during March–June 2020 comprehensive questionnaire about the adoption and perception of EHRs. 67 anaesthesiologists/physician responses	About 88% of respondents strongly agree or agree that EHRs positively changed their working clinical experience. EHRs are positively affecting healthcare cost, quality of care, communication, patient satisfaction, efficiency of practice and occurrence of medical errors. However, the respondents are about evenly divided regarding the burnout because of the use of EHRs. More than 75%– 100% agree that the given features are either very easy or at least easy to use. The small deviation from the ‘near consensus’ is seen in the answers about the continuity of care (67%) and analysis of care results (69%) which is not a bad result in general at least 67% agreement regarding the consistency of monitored and recorded clinical details and suitability of documentation features, while the screens and layouts of pages and other web-based tools found an agreement of 69%.	The above results show that the young generations of specialists (86%) are more adaptable to change and accept the EHRs even if the experience they have is less than three years. the need for integrating some EHRs related training in the curriculum of medical schools is of paramount importance
17-AlSadrah, Sana (22)	Electronic Medical Records and Health Care Promotion in Saudi Arabia	In this literature review, the author focused on the benefits of widespread adoption of EMRs in Saudi Arabia, the perceptions of health care professionals, and the challenges and barriers toward improved implementation of this technology.	The ease of communication is another major advantage when health care professionals can communicate with patients through email, fax, and phone.18 important for clinical investigations, but for evaluating health care policies and informing stakeholders about approaches to improve access to high-quality health care.25 Other advantages related to accessibility and management include management and records of patient referrals, allowing health care professionals, even when out of the hospital, to access patient health records, allowing patients to access parts of their health records and providing data backup and disaster recovery.2	Recommendations are related to improving the communication between different health care personnel as well as between physicians and their patients. Ensuring the possibility of short message service (SMS) or email communication is a must. This can be achieved by providing quality Internet connection to hospitals and other healthcare delivery facilities, improving patients’ awareness about the value of online communication with their health care provider, and increasing physicians’ computer literacy.39 Physicians should also be able to customize their preferred, easy-to-use EMR interface and should be able to select the optimal tools to achieve effective communications with their patients.40 A literature review summarized the barriers of EHRs by physicians and classified them into 8 main categories. These categories included technical, financial, psychological, social, legal, time, organizational, and change processes. In short, the main highlighted barriers across the eight categories were probable security breaches, loss of access to data upon computer crashes or power failures, the time needed to enter data and check their quality, the complexity of the technology (especially among personnel with poor English language and computer skills), the potential to disturb physician-patient communication, and the lack of system customization for all hospital needs. Other barriers were concerned about the lack of continuous support from IT staff in hospitals and poor training of health care personnel in the use of EMR.
18-Koutzampasopoulou Xanthidou (23)	Electronic Medical Records in Greece and Oman: A Professional's Evaluation Of Structure And Value	The study took place in the natural setting of the medical units’ environments. A purposive sample of 40 professionals in Greece and Oman, was interviewed.	Some believe that religion should be included in the patient's record to help understand the patient's habits and, possibly, culture that affect an individual's health condition while others find such an idea highly disputable, to say the least, pointing to the discrimination practices that it might lead to. Likewise, with the cases of a person's nationality, language, and religion; some find the idea of their inclusion to the EMR useful or even essential while others find it unacceptable. Category of ‘eating habits’ or ‘life style’—MD could provide better health care to the patients and avoid mistakes that are related to the elimination of the effects of a medicine because of these behaviors. The race, education level and the language are important because they may assist in communicating with the patient.”	The study suggests that: (1) The demographics of the EMR should be divided in categories, not all of them accessible and/or visible by all; (2) The EMR system should follow an open architecture so that more categories and subcategories can be added as needed and following a possible business plan (ERD is suggested); (3) The EMR should be implemented gradually bearing in mind both medical and financial concerns; (4) Sharing should be a patient's decision as the owner of the record. Reaching a certain level of maturity of its implementation and utilization, it is useful to seek the professionals’ assessment on the structure and value of such a system.

## Results

Figure 1 shows the number of studies identified, excluded (duplication, not EHR-focused), and selected with the criteria for the systematic review. This process generated 18 eligible articles.

### Study characteristics

A summary of the results found for each of the 18 articles is provided in Table 1 (6–24). The articles that met the inclusion criteria were assessed to determine the characteristics and effect of EHR implementation in GCC countries, including a summary of the study features such as information on doctor communication, quality of care, medication error, data retrieval and waiting time. The Newcastle-Ottawa Scale (NOS) was applied for quality assessment, as shown in Table 2. Publication dates ranged from 1992 to 2021. The number of participants in each study varied from 23 to 700, including male and female healthcare workers.

**Table 2:** The New-Ottawa scale (NOS).

NOS criteria	Studies (year)																	
	1	2	3	4	5	6	7	8	9	10	11	12	13	14	15	16	17	18
	Al-Farsi (6)	Al-Azmi et al. (7)	Al-Mujaini et al. (8)	Al-Jafar (9)	Al-Rowibah et al. (10)	Al Alawi et al. (11)	Alasmary et al. (12)	Alharthi et al. (13)	Shaker et al. (14)	Bani-Issa et al. (15)	Alsohime et al. (16)	Al-Hashimi et al. (17)	Khalifa (18)	Saddik and Al-Fridan (19)	Wali, R. M. 2020	Jabali, A Karim 2021	AlSadrah, Sana A et al, 2020	Xanthidou, Ourania 2018
A. Selection (maximum of four points)																		
1. Representativeness of the exposed cohort	1	1	1	1	1	1	1	1	1	1	1	1	1	1	1	1	1	1
2. Selection of the non-exposed cohort	1	1	1	1	1	1	1	1	1	1	1	1	0	1	1	1	1	1
3. Ascertainment of exposure	1	1	0	0	0	0	0	1	1	1	1	0	0	1	0	1	1	0
4. Demonstration that outcome of interest was not present at start of study (no bone disease at start of study)	1	1	1	1	1	0	1	1	1	1	1	1	1	0	1	1	1	1
B. Comparability (maximum of two points)																		
1. Confounding factors	1	1	1	1	1	1	1	1	1	1	1	1	1	1	1	1	1	1
C. Outcome (maximum of three points)																		
1. Assessment of outcome	1	1	1	0	1	1	1	1	1	1	1	1	1	1	1	1	1	1
2. Statistical	1	1	1	1	1	1	1	1	1	1	1	1	1	1	1	1	1	1
Total (maximum of nine points)	7	7	6	5	6	5	6	7	7	7	7	6	5	6	6	7	7	6

### Effect of EMR on quality of care

Almost two-thirds of the studies (72%) found that EHR improves the quality of care for patients, noting that retrieval and entry of patient information is more straightforward and more accurate, access to the system is efficient, and the presence of an EHR reduces medical errors and enhances work productivity (6, 7, 10, 14, 16, 18–24) and comfort while entering the data through typing instead of writing (14). This general finding supports the previously shown positive impact of advanced technology in the healthcare sector and supports the desire of GCC healthcare providers to adopt paperless systems (10, 17). Nevertheless, lack of reliability or completeness of data (13, 15) and gaps in users' computer skills (15, 17) are also reported.

### Effect of EHR on doctor-patient relations

The disadvantages of EHRs listed in the assessed publications included concerns about confidentiality (6, 11), general underutilisation of the system (6, 7), and a need for applicable or flexible disease coding (6). In Kuwait (7, 9), the participants disliked the requirement for physicians to work with the computer in the examination room. In Al Alawi's study in the United Arab Emirates (UAE), this behaviour was perceived as reduced attention and communication, lack of eye contact and added overall waiting time (11). Increasing difficulty with work performance after applying the EHR system has also been reported (8). For example, it has been mentioned that the system was inadequate (24), slow (6, 19) or lacked a user guide during medication ordering (10).

## Discussion

This review investigates the impact of EHR systems on the relationship between doctors and patients in the Gulf countries. Our results showed that the EHR facilitates the development of an environment in which all relevant patient clinical data is captured, which in turn helps doctors to make decisions that directly influence patient outcomes. Furthermore, in agreement with other literature, it has been observed that EHR facilitates communication between patients and doctors (5, 25). The enhanced communication between patients and doctors improves the overall quality of care delivery in healthcare settings, especially in the physician-focused healthcare systems in Gulf countries.

However, it has been observed that some patients would like more satisfaction with respect to the implementation of EHR in the healthcare setting. There is a sense that doctors shift attention away from their patients and towards the recording of their entries. Therefore, patients feel dissatisfied, feeling doctors require more time for “data entry” than they do to assess the presenting patient.

Implementation of EHR was described as improving the transmission of accurate and reliable patient information, reducing chances of medication errors and “lost files”. Furthermore, it was observed that the use of EHR improves the ability of physicians to note relevant information at the time of consultation rapidly. This, in turn, allows them to invest more time in decisions relating to medication and treatment, thereby improving the quality of care delivered to the patients. Despite this, it should also be noted that the implementation of EHR leads to large changes in physician workflow and processes, which may lead to increased errors during this transition period and thus adversely affect patient outcomes. As revealed in this study, the introduction of EHR systems brings about significant changes in the daily routines of healthcare professionals. These transitional challenges may contribute to a temporary increase in errors during the initial stages of implementation, necessitating the need for comprehensive change management strategies. It is imperative that healthcare organisations pay close attention to mitigating these disruptions to maintain the quality of patient care.

Furthermore, it is noteworthy that while EHRs are demonstrated to enhance healthcare delivery in the Gulf countries overall, there exists a divergence in perspectives between healthcare providers and patients. Physicians often perceive the benefits of EHRs, such as improved data accuracy and efficiency. However, patients may feel that these systems lead to reduced attention from their doctors, lack of eye contact, and extended waiting times. Addressing this disparity in perceptions through comprehensive education and transparent communication is pivotal to ensuring that the full potential of EHR systems is realised while simultaneously upholding patient satisfaction and trust in the healthcare system.

These behavioural outcomes are generally consistent with those previously observed in systematic Western healthcare facility reviews (5, 25–29). Similarities to this study include that effective communication of the benefits of EHRs to patients needed to be improved, leading to a general impression that physicians were spending more time on their computers than with the patients themselves. But that, nonetheless, led to enhanced surveillance and monitoring and decreased medication errors. Hence, it may be generally agreed that the benefits of EHRs are not sufficiently communicated to all stakeholders. Even though all the studies were of good quality according to the NOS, limitations of this study included a lack of data and publications from all Gulf countries on this topic. Future studies should tackle the EHR and patient-healthcare provider relationship to further explore the impact of digital transformation in health systems.

### Recommendations

Through this review, it has been noted that there needs to be more computer skills and trust in the EHR system, which leads to problems in effective implementation. This, in turn, hampers the potential relationship between doctors and patients in Gulf countries. The authors provide the following suggestions to ensure the effective implementation of EHR in healthcare settings of Gulf countries:

• Physicians should be provided with specific training on the EHR system being implemented, preferably paired with general computer training in medical school and during the early years of the residency programmes. This is essential considering the huge surge of artificial intelligence and efforts to digitalise health systems.
• Trust in the system itself should be built by openly communicating the objectives and advantages of an EHR system to physicians. In addition, the system implementation outcomes should also be communicated by doctors to patients. This will help to improve patient perspectives of the change and reduce overall dissatisfaction.

## Conclusion

Implementing EHR systems is beneficial for effective care delivery by doctors in the Gulf countries. EHR assists doctors with recording patient details, including medication and treatment procedures, as well as their outcomes. Despite the patient's perception of decreased attention, EHR promotes and enhances healthcare delivery in the GCC. The EMR has the potential to be a key tool for improving continuity of care in the GCC by flagging up follow-up dates and tests, medications, and overall surveillance. Based on this study, the authors conclude that widespread EHR implementation is highly recommended. However, specific training should be provided, and all users' subsequent effects on adoption rates must be evaluated (particularly physicians). The COVID-19 pandemic showed the great value of EHR in accessing information and consulting patients remotely.

## Data Availability

The original contributions presented in the study are included in the article/Supplementary Material, further inquiries can be directed to the corresponding author.
